# Fluid biopsy for circulating tumor cells in an occult ovarian cancer patient exhibiting bilateral supraclavicular lymph node metastases: A case report

**DOI:** 10.3892/ol.2013.1364

**Published:** 2013-05-24

**Authors:** SHIZHI HE, PINGDONG LI, XIAOHONG CHEN, ZHENKUN YU

**Affiliations:** Department of Otolaryngology-Head and Neck Surgery, Beijing Tongren Hospital, Key Laboratory of Otolaryngology-Head and Neck Surgery, Ministry of Education, Capital Medical University, Beijing 100730, P.R. China

**Keywords:** head and neck cancer, unknown primary tumor, supraclavicular lymph node metastases, ovarian cancer, circulating tumor cells

## Abstract

Metastases to the supraclavicular region usually originate from the head and neck or from infraclavicular tumors. Ovarian primaries of supraclavicular metastases are extremely rare. The present study reports the case of a 60-year-old patient with a bilateral supraclavicular mass that was diagnosed as a poorly-differentiated squamous cell carcinoma of unknown primary, following a fine-needle aspiration biopsy (FNAB) and comprehensive clinical investigation. The analysis of a peripheral blood sample using the CellSearch system revealed the presence of circulating tumor cells (CTCs) that were positive for epithelial cell adhesion molecule (EpCAM) and cytokeratin (CK) expression. Since EpCAM is usually expressed in adenocarcinoma, an excisional biopsy of the right supraclavicular lymph node was performed. The patient was diagnosed with occult ovarian low-grade serous carcinoma by immunohistochemistry. To the best of our knowledge, this is the first report to demonstrate that CTCs may be detected in the peripheral blood of a patient with cancer of unknown primary (CUP) by using the CellSearch system. A literature review was performed to analyze the diagnostic procedures of CUP metastatic to the cervical lymph nodes and the clinical features of CTCs.

## Introduction

Metastases to the supraclavicular region may originate from the head and neck and from infraclavicular tumors. The principal metastatic sites from the head and neck region are the hypopharynx, oropharynx and nasopharynx. Infraclavicular primary sites mostly originate from the lung, breast, lower digestive tract and genitourinary tract (1). Ovarian malignancies are rare primary sites for supraclavicular lymph node metastases. The present study reports a case of an occult ovarian cancer patient with bilateral supraclavicular lymph node metastases, where positive circulating tumor cells (CTC) were detected by the CellSearch system at the time of diagnosis. To the best of our knowledge, this is the first study to demonstrate that CTCs may be identified in patients with cancer of unknown primary (CUP) using the CellSearch system. In addition, the diagnostic procedures of CUP metastatic to the cervical lymph nodes and the clinical features of CTC are discussed with regard to the current knowledge.

## Case report

**Figure 1.**
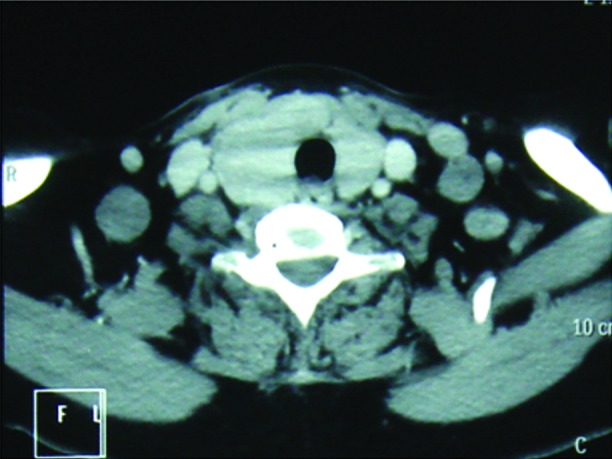
Computed tomography (CT) scan of the neck showing bilateral supraclavicular lymphadenopathy.

In February 2011, a female 60-year-old Chinese patient was admitted to the Otolaryngology-Head and Neck Surgery Department of the Beijing Tongren Hospital (Key Laboratory of Otolaryngology-Head and Neck Surgery, Ministry of Education, Capital Medical University, Beijing, China) complaining of a growing mass in the right side of the neck that had been present for six months. No other symptoms were observed. The patient’s surgical, medical and family histories were unremarkable. In the ear-nose-throat examination, a 2×2.5 cm solid, fixed mass was identified in the right supraclavicular fossa and multiple 1×1.5 cm solid masses were observed in the left supraclavicular fossa. A fine-needle aspiration biopsy (FNAB) was performed in the right supraclavicular mass and the pathological diagnosis was of a metastatic poorly-differentiated squamous cell carcinoma. Subsequently, the patient underwent a detailed comprehensive examination of the head, neck and upper aerodigestive tract using nasolaryngoscopy, bronchoscopy and gastroenteroscopy and CT scans of the neck and chest for primary malignancy, which demonstrated only lymphadenopathy in the bilateral supraclavicular region (Fig. 1). The patient was, therefore, diagnosed with metastatic cervical carcinoma of unknown primary.

**Figure 2.**
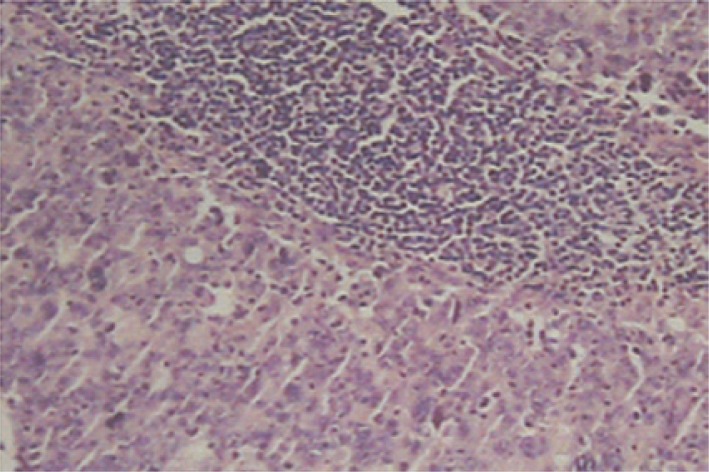
Histopathological examination (haematoxylin and eosin staining; magnification, ×100) of the right supraclavicular lymph node showing poorly differentiated adenocarcinoma.

**Figure 3.**
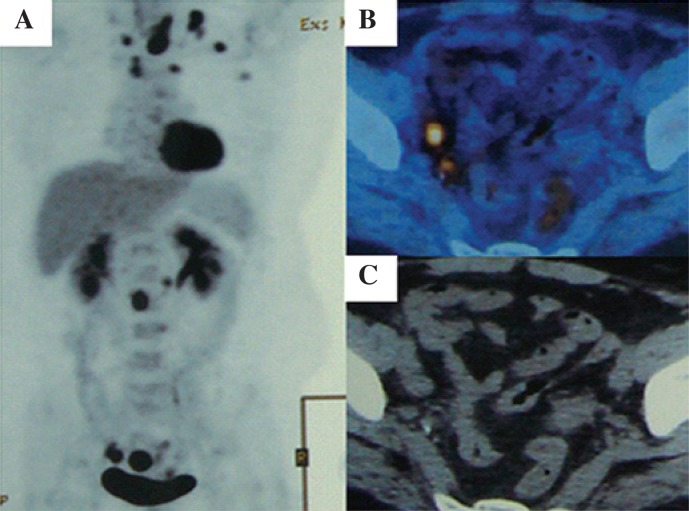
Whole body positron emission tomography (PET) scan combined with computed tomography (CT). Coronal maximum intensity projection image revealing (A) intense FDG uptake in the bilateral supraclavicular lymph nodes. PET/CT image (B) and CT image (C) revealing an enlarged right ovary lesion with intense FDG uptake. FDG, ^18^F-fluorodeoxyglucose.

Once informed consent had been obtained, a 7.5-ml peripheral blood sample was collected from the patient and then analyzed with the CellSearch system as described previously (2). Three positive circulating tumor cells (CTCs) were identified with positive expression for epithelial cell adhesion molecule (EpCAM) and cytokeratin (CK)8, 18 and 19. Since EpCAM is usually expressed in adenocarcinoma, an excisional biopsy of the right supraclavicular lymph node was performed. The findings supported the diagnosis of a poorly-differentiated, metastatic adenocarcinoma (Fig. 2). Immunohistochemistry revealed that the specimen tested positive for cancer antigen (CA)-125, cytokeratin (CK)8/18, CK7, estrogen receptor (ER) and carcinoembryonic antigen (CEA), indicating a possible gynecological origin for the metastasis. Negative reactions were observed for thyroid transcription factor (TTF)-1, CK20, CK5/6, Epstein-Barr virus, vimentin, neuron specific enolase (NSE), S-100 protein, gross cystic disease fluid protein (GCDFP)-15, thyroglobulin (TG), calcitonin and the progesterone receptor (PR). The serum CA-125 level was elevated to 1,028 U/ml (normal <35 U/ml). ^18^F-fluorodeoxyglucose (FDG) positron emission tomography combined with computed tomography (PET-CT) revealed an intense uptake of FDG in the bilateral supraclavicular lymph nodes and a right pelvic mass with a diameter of 3.9 cm (Fig. 3).

The patient was referred to the Gynecology Department for further therapy. The patient underwent a bilateral salpingo-oophorectomy and cytoreductive surgery and an ultimate diagnosis of ovarian low-grade serous carcinoma [International Federation of Gynecology and Obstetrics (FIGO) Stage IV] was established by histological examination. Subsequent to surgery, the patient was treated with 135 mg/m^2^ paclitaxel on day 1 and 75 mg/m^2^ cisplatin on day 2 intravenously, continuing at 3-week intervals. The patient succumbed to the disease six months later due to disease progression.

## Discussion

CUP is defined as the histological diagnosis of metastasis without the detection of a primary tumor. CUP metastatic to the cervical lymph nodes accounts for ∼3–5% of all head and neck cancers (3). Squamous cell carcinoma (SCC) is the most common histology, representing 65% of cases, followed by undifferentiated carcinoma (22%) and adenocarcinoma (13%) (4). However, in the case of supraclavicular node metastases, 50–76% patients have adenocarcinoma (5). Patients with adenocarcinoma in the metastatic lymph nodes usually have a primary lesion located outside of the head and neck area, including in the lung, breast, lower digestive tract or genitourinary tract (6). The location of the lymph node may indicate the site of origin of the primary tumor. For example, the presence of left supraclavicular lymphadenopathy, ‘Virchow’s node’, has been regarded as indicating the presence of cancer in the gastrointestinal tract. By contrast, right supraclavicular lymphadenopathy indicates the presence of cancer mainly from the lungs, esophagus and mediastinum.

The main objectives of the diagnostic evaluation of a patient with CUP are to determine the histology of the metastatic tumor and identify the primary tumor. Following a routine detailed physical examination of the head and neck and of the upper aerodigestive tract using panendoscopy, the initial tissue diagnostic procedure is a FNAB (7). A FNAB results in a representative cellular sample in the majority of CUP patients. A diagnosis is usually established with routine histological staining, supplemented with immunochemistry, achieving a diagnostic sensitivity of 83–97% and a specificity of 91–100% for metastatic lesions (1)

In the majority of cases, the biological material obtained by FNAB is sufficient for diagnosis. However, in certain cases, insufficient immunophenotypic characterization of the available biological material may lead to misdiagnosis (8). For example, poorly-differentiated adenocarcinoma and carcinoma may be observed with descriptions of the same spectrum of histological appearance, with carcinoma cells showing a lesser degree of glandular differentiation. However, a subset of patients with poorly-differentiated adenocarcinoma may be distinctive in their tumor biology and responsiveness to radiotherapy and chemotherapy. For the last few years, CTCs have received significant attention as alternative markers (9). It has long been hypothesized that the levels of CTCs in the peripheral blood correlates with the aggressiveness of the tumor (10). The detection and characterization of these cells is likely to significantly improve the early detection of tumor spreading. The molecular characterization and specific biological properties of CTC may provide important diagnostic information, including the assessment of the tumor of origin (11). Furthermore, case reports have suggested that the morphological features of CTCs resemble the corresponding primary and/or metastatic lesions in the breast (12). CTCs may be used to survey primary and metastatic lesions through minimally invasive peripheral blood draws or ‘fluid biopsies’.

In the present study, it was shown that the analysis of peripheral blood using the CellSearch system provided additional information on the immunophenotype of the tumor cells. To the best of our knowledge, the present study is the first to demonstrate that CTCs may be identified in patients with CUP. This technology uses positive selection with magnetically labeled anti-EpCAM and immunocytochemical staining for CK8, 18 and 19 to isolate and enumerate CTCs. In this regard, the expression of EpCAM has been shown to occur in almost all adenocarcinomas, including ovarian adenocarcinomas (13,14).

The CellSearch system has been approved by the US Food and Drug Administration (FDA) and is the only validated and standardized CTC detection system to be introduced into clinics. Increasing evidence suggests that CTC numbers are an independent predictor of progression-free survival (PFS) and overall survival (OS) in breast, colorectal and prostate cancer according to the CellSearch system (15). Poveda *et al* reported a multicenter, randomized, exploratory study that included 216 patients with relapsed/recurrent advanced ovarian cancer (16). The study observed that 45% (97/216) of the patients were CTC-positive and that the patients with ≥2 CTCs at baseline had a significantly shorter overall survival time and time to disease progression compared with CTC-negative patients. However, the use of the CellSearch system to identify CTCs in CUP has not been studied at this time.

During the last two decades, PET and PET-CT have been increasingly used in diagnostic procedures for CUP. Several studies have evaluated the ability of PET-CT to detect primary tumors in patients with cervical lymph node metastases of unknown origin. Rusthoven *et al* published a meta-analysis based on 16 studies that included 302 patients with cervical lymph node metastasis from an unknown primary (17). PET detected the primary tumor in 25% of the patients in whom panendoscopy and CT failed to identify a primary tumor. Kwee and Kwee published a meta-analysis with 11 studies and 433 CUP patients analyzed using PET-CT (18). The global rate of tumor detection was 37%, with a sensitivity and specificity of 84%. Al-Ibraheem *et al* analyzed another set of eight studies from between 2000 and 2009, in which PET or PET-CT was used in 180 patients with cervical lymphadenopathy of unknown origin. The study reported a 28.3% detection rate for primary tumors with 37% false-positive scans (19). Accordingly, a recent interdisciplinary consensus conference stated that PET-CT may be regarded as a procedure in the diagnostic workup of CUP patients (7). However, it is essential to be aware of the limitations and drawbacks of PET-CT, including the high rate of false-positive findings, the limited availability of the procedure, the costs and the burden to the patient. Further studies are required to assess the value of this procedure when weighed against other diagnostic techniques.

Ovarian cancer is the second most common gynecological cancer and the leading cause of mortality from gynecological malignancy. The distant metastasis of ovarian cancer mostly involves the liver, lung and bone. Lymphadenopathy in the neck is an unusual presentation of malignant neoplasms of the ovary and may occur prior to there being evidence of an ovarian mass; their detection may represent a challenge for the oncologist (20). Only a few cases of neck metastases associated with ovarian malignancies have been reported. Table I shows a summary of case reports for ovarian tumors with bilateral supraclavicular metastases from the English-language literature. In a series of 100 autopsies on female patients who succumbed to ovarian carcinoma, the incidence of extra-abdominal lymphadenopathy in the supraclavicular lymph nodes was shown to be only 4% (21). In a review of 35 patients with extra-abdominal lymphadenopathy of ovarian cancer, 11 patients were demonstrated to exhibit supraclavicular metastases (22). However, no patient exhibited bilateral supraclavicular metastases such as that shown in Table I.

Clinically, a differential diagnosis in terms of cervical adenocarcinomas of unknown primary is that of metastases from the lung, breast and gastrointestinal tracts. Immunohistochemical findings are crucial in forming a differential diagnosis. Communication between the pathologist and clinician is extremely important for defining the tumor origin (4). There are a number of relatively specific tumor markers that may aid in the identification of the site of the cancer. CK7 and CK20 are the most common CK strains used for identifying adenocarcinomas. CK7 is detected in tumors of the lung, ovary, endometrium and breast, but not in lower gastrointestinal tract tumors. CK20 is normally expressed in the gastrointestinal epithelium, urothelium and Merkel cells (23). The CK phenotype CK20^+^/CK7^−^ markedly favors colonic primary tumors. Certain studies have reported that 75–95% of colon tumors are CK20^+^/CK7^−^, while ∼85% of lung carcinomas are CK20^−^/CK7^+^(24). TTF-1 is another lung cancer marker and one that ∼68% of adenocarcinomas and 25% of squamous cell lung carcinomas have been shown to stain positively for (25). GCDFP-15 is an apocrine differentiation marker and is specifically expressed in patients with breast carcinomas. Among the patients with breast carcinomas, 62–72% have been identified as GCDFP-15-positive (26).

The main positive markers for ovarian adenocarcinomas are ER, PR, CA-125 and Wilm’s tumor 1 (WT1). ER and/or PR are positive in 50–83% of ovarian serous carcinomas. Carcinomas arising in the breast and other sites in the female genital tract are also ER- and PR-positive, whereas carcinomas from other locations are ER- and PR-negative (27). WT1 and CA-125 are expressed in the majority of ovarian serous carcinomas, but the majority of ovarian clear cell and mucinous carcinomas are negative for the markers (28). CA-125 is also expressed in a minority of carcinomas, including those of the breast, endometrium, cervix and lung. The expression of WT1 is usually negative in breast, gastrointestinal and pancreatobiliary primaries.

In conclusion, to the best of our knowledge, the present study is the first to demonstrate that CTC may be detected in the peripheral blood of patients with CUP when using the CellSearch system. The molecular characterization and specific biological properties of CTC may provide important information for the diagnosis of CUP. Further studies on a larger patient population are required to evaluate the significance of CTCs as diagnostic decision markers in CUP. Although ovarian cancer rarely metastasizes to the cervical lymph node, it should be considered in the differential diagnosis of lymphadenopathy in the supraclavicular area of postmenopausal individuals.

## Figures and Tables

**Table I.** Cases of ovarian cancer with bilateral supraclavicular lymph node metastases reported in the English-language literature.

First author, year (ref.)	No. of cases	Pathology	FIGO stage	Treatment	Follow-up (years)
Malpica *et al*, 2001 (29)	1	LGS Ca	IB	S+C	NED (6.0)
Verbruggen *et al*, 2006 (30)	1	Serous borderline ovarian tumor	IV	S+C	NED (4.5)
Present study	1	LGS Ca	IV	S+C	STD (0.5)

STD, succumbed to disease; NED, no evidence of disease; LGS Ca, low-grade serous carcinoma; S, surgery; C, chemotherapy; FIGO, International Federation of Gynecology and Obstetrics.
